# Computerized Clinical Database Development in Oncology

**DOI:** 10.4103/0973-1075.76229

**Published:** 2011-01

**Authors:** SVS Deo

**Affiliations:** Department of Surgical Oncology, IRCH All India Institute of Medical sciences, New Delhi, India

**Keywords:** Health informatics, Oncology, Computerized clinical data base

## Abstract

In the era of evidence based medicine documentation of clinical data is extremely important. The field of Health informatics is a discipline at the intersection of information science, computer science and health science. Current health informatics field is mainly catering to the general needs of hospital setups. Development of disease / organ/ specialty based computerized clinical data base is still in its infancy and there is a need for clinicians to actively involve in this field to generate authentic and analyzable clinical data. In this article we present our experience of computerized oncology clinical data base development.

During the last three decades, the practice of medicine has witnessed major changes. There is an exponential increase in medical research, and clinical practice patterns are also changing rapidly all over the world. The current treatment guidelines are mostly based on evidence. In the era of “Evidence based medicine,” data documentation plays a major role. During the same period, developments in the field of computer sciences and bioinformatics have revolutionized medical data documentation and analysis.

However, currently, the majority of the evidence for formulating treatment guidelines originates from western countries, and clinicians from developing countries follow those treatment guidelines without critically evaluating the feasibility, applicability and efficacy of such protocols in the setting of developing countries. There is an urgent need to create a clinical database of cancer patients in developing countries to address these issues.

Currently, general purpose ready-to-use software for multispecialty hospitals are available and are being used for patient registration, billing, medical stores, pharmacy and limited out-patient and in-patient activities. These commercial softwares have severe limitations as far as the clinical and academic needs are concerned. The needs of a clinician with dedicated academic oncology practice are different. A custom-built, detailed and structured clinical database can only cater to such academic needs.

The clinician plays a crucial role in the planning, development and execution of a clinical database program. He has to have an in-depth understanding of the specialty and the anticipated needs from such database. The process of such software development involves extensive brainstorming with other team members, including clinical colleagues and computer engineers. During the process of development, the clinical database can be divided into small workable modules like: (1) Demographics, (2) Clinical characteristics, (3) Investigations, (4) Treatment details, (5) Treatment outcomes, (6) Other outputs. Each of these modules should contain 10–15 structured, semi-structured or open-ended queries. Attempts should be made to create queries that are directly analyzable and minimize open-ended queries. You can plan and generate a number of additional output modules like census, discharge summary, morbidity and mortality, etc. The database can be designed as a broad-based oncology database or a disease-specific database if you are engaging in organ- or disease-specific sub-specialization. For example, for a palliative care practice, the focus of the database should be on symptom type, severity and duration, treatment intervention and outcome scales. The basic program can be developed on a standard and widely available software platform like MS access, and a program like Visual basics can be used as a front end. Once the software is created, data entry should be done as part of routine clinical work by the clinical team rather than delegating the job to data entry operators, to minimize errors and for maintaining quality control. Regular audit of the database for checking entries and taking a backup is extremely crucial. Once the data reaches a critical mass, the data can be transferred to analytical softwares like SPSS for outcome analysis easily.

In a small/solo practice setting, a standalone computer can cater to the needs, whereas in a bigger setup, networking can be planned. The major advantages of a computerized database are ready availability of the data for census, audit, identify patient profile, treatment patterns, outcome analysis and last, but not the least, ready availability of clinical data for academic presentations and publications. In the long term, these databases can provide material for planning major epidemiological and intervention trials.

(The author was involved in creating a Computerized clinical data base for the department of Surgical Oncology, BRA-IRCH, AIIMS, New Delhi, which currently contains complete demographic, clinical, treatment and outcome details of more than 6000 cancer patients.)

